# Head and Neck Rhabdomyosarcoma in Children: An Otolaryngological Perspective on Diagnostic and Therapeutic Approaches

**DOI:** 10.1007/s11912-026-01823-1

**Published:** 2026-08-25

**Authors:** Agata Wojno, Milena Chmielewska, Sara Shefa, Oliwia Cichy, Paulina Lenkiewicz, Karolina Dorobisz

**Affiliations:** 1 Student Scientific Club of Otolaryngoly, Faculty of Medicine, Wroclaw Medical University, Wroclaw, 50556 Poland; 2 Department and Clinic of Otolaryngology, Head and Neck Surgery, Wroclaw Medical University, 50556 Wroclaw, Poland; 3 Department of Otolaryngology, Wroclaw Medical University, Łąkowa 63, Krotoszyn, 63-700 Poland

**Keywords:** Rhabdomyosarcoma, Pediatric oncology, Head and neck neoplasms, Otolaryngology, Multimodal therapy, FOXO1 fusion

## Abstract

**Purpose of Review:**

Head and neck rhabdomyosarcoma (HNRMS) is the most common soft-tissue sarcoma in children, with approximately 30–40% of cases arising in the head and neck region. Due to its anatomical complexity, histopathological diversity, and molecular heterogeneity, HNRMS remains a major diagnostic and therapeutic challenge in pediatric otolaryngology. This review aims to summarize current evidence regarding the epidemiology, clinical presentation, diagnostic strategies, contemporary treatment modalities, and long-term complications associated with pediatric HNRMS. Purpose of Review.

**Recent Findings:**

Recent advances in molecular pathology have significantly improved risk stratification in rhabdomyosarcoma, particularly through the identification of FOXO1 fusion status as a key prognostic marker. Modern imaging techniques, especially magnetic resonance imaging and PET-based assessment, have enhanced diagnostic precision and evaluation of local tumor extension. Contemporary multimodal treatment protocols combining chemotherapy, conservative surgery, and proton beam radiotherapy have improved survival outcomes while reducing treatment-related morbidity. Furthermore, growing attention has been directed toward survivorship issues, including craniofacial deformities, endocrine dysfunction, hearing impairment, and secondary malignancies.

**Summary:**

The prognosis of pediatric HNRMS depends strongly on tumor location, histological subtype, and molecular characteristics. Orbital tumors are associated with favorable outcomes, whereas parameningeal lesions continue to pose substantial therapeutic difficulties because of delayed diagnosis and proximity to critical anatomical structures. Early recognition, accurate histopathological and molecular diagnosis, and individualized multidisciplinary treatment are essential to optimize survival and minimize long-term complications. Ongoing developments in targeted therapy and proton radiotherapy may further improve both oncological outcomes and quality of life in affected children.

This review is important because it integrates current molecular, diagnostic, and therapeutic advances in pediatric HNRMS from an otolaryngological perspective, highlighting both survival outcomes and long-term quality-of-life considerations. Moreover, it emphasizes the need for early multidisciplinary management in anatomically complex head and neck tumors, which remains crucial for improving prognosis and reducing treatment-related morbidity.

## Introduction

Rhabdomyosarcoma (RMS) is a malignant soft-tissue neoplasm of mesenchymal origin, exhibiting variable features of differentiation toward skeletal muscle [1–3]. It is the most common soft-tissue sarcoma of childhood, as well as one of the significant extracranial solid tumors in children [1, 4, 5] The proportion of RMS among soft-tissue sarcomas is approximately 50% [2, 3], and within the overall spectrum of childhood cancers it accounts for 3–5% of malignant neoplasms in children [1, 5–7]. Moreover, the proportion of RMS located within the head and neck region is approximately 30–40% of pediatric cases, or about 40% of all RMS cases [3, 8–11].

Its clinical significance results primarily from the marked heterogeneity of its anatomical location, histology, and molecular profile, which directly affects treatment stratification and assessment of the risk of recurrence [8, 12]. From an otolaryngological perspective, the significance of this localization stems from the presence of tumors within the nose, nasal cavities, paranasal sinuses, nasopharynx, middle ear, mastoid process, skull base, pterygopalatine and infratemporal fossae, as well as non-parameningeal structures such as the salivary glands, tongue, or palate [3, 8]. From an otolaryngological perspective, head and neck RMS represents a particular clinical challenge, because tumors may develop in areas typically assessed and treated by an otolaryngologist, including the nose and nasal cavities, paranasal sinuses, nasopharynx, middle ear, mastoid process, skull base, pterygopalatine and infratemporal fossae, as well as in non-parameningeal sites such as the salivary glands, tongue, or palate [3, 8].

Thus, the aim of the present review is to present current diagnostic and therapeutic methods used in children with head and neck RMS, with particular emphasis on issues relevant to otolaryngology: the recognition of lesions in complex anatomical sites, histopathological and immunohistochemical confirmation, the role of local treatment, and the long-term consequences of therapy within the craniofacial region and the organs of the head and neck.

### Epidemiology and Etiopathogenesis

Morfouace et al. [13] reported that patients with Head and Neck Rhabdomyosarcoma (HNRMS) are usually diagnosed early, with a median age of 5 years, while Krommenhoek et al. [7] indicated that nearly 70% of HNRMS cases manifest before the age of 10 years, with a mean age at diagnosis of 5 years, and the peak incidence is reported between 3 and 5 years of age [8]. In an international genomic analysis of RMS, the median age was 5.9 years, with a range from 0.02 to 37.8 years [4]. In the EpSSG study concerning non-parameningeal head and neck RMS, the median age was 6.4 years, with a range of 0.1–25 years [5]. In a Polish study, the median age was 4.8 years [14].

Histologically, RMS comprises four main subtypes: embryonal RMS (ERMS), alveolar RMS (ARMS), spindle cell/sclerosing RMS, and pleomorphic RMS, with ERMS and ARMS predominating in the pediatric population [9, 14, 15]. In the pediatric population, the embryonal and alveolar forms predominate [16], and the proportion of individual subtypes depends on the characteristics of the cohort studied, particularly age range, tumor site, and whether the cohort includes all pediatric RMS cases or is restricted to head and neck/parameningeal RMS and molecularly characterized tumors. Thus, in broadly defined pediatric RMS cohorts, ERMS is usually the most frequent subtype, whereas cohorts restricted to head and neck RMS may show a relatively higher proportion of ARMS. Gallagher et al. [9] reported ARMS as the most common subtype (43.2%) in a molecularly characterized HNRMS cohort, in which parameningeal tumors predominated (57.7%). Frankart et al. [8] report that ERMS accounts for approximately 70% of pediatric RMS cases and includes the botryoid and spindle-cell variants, whereas ARMS accounts for approximately 20% of pediatric cases. In the study by Radzikowska et al. [14] which included a pediatric population, ERMS was diagnosed in 61% and ARMS in 39% of patients. In a population-based analysis of HNRMS in children and adolescents, Wu and Zeng [17] identified ERMS in 65.9%, ARMS in 22.7%, and other types in 11.4% of patients. In the analysis by Rohde et al. [10], encompassing all age groups, ERMS was also the most common subtype, accounting for 45% of cases, followed by ARMS at 32%. Nevertheless, it was noted that among pediatric patients the proportion of ERMS was the highest, whereas the percentage of ARMS increased in the 18–39 and 40–64 age groups [10].

The etiopathogenesis of RMS remains incompletely elucidated; however, in the literature it is described as a malignant proliferation with a skeletal muscle phenotype, arising from immature mesenchymal cells or primitive embryonal cells, with features of impaired myogenic differentiation [2, 3, 8]. Most cases are sporadic; however, Frankart et al. [8] indicate an association of RMS with Beckwith–Wiedemann, Costello, and Li–Fraumeni syndromes, while Zhen et al. [18] emphasize the association of Li–Fraumeni syndrome with germline mutations in the TP53 tumor suppressor gene and the need to assess family history in the context of cancer predisposition.

In the molecular pathogenesis of RMS, the FOXO1 fusion status is of fundamental importance, as it biologically distinguishes FOXO1-positive tumors from FOXO1-negative tumors. In alveolar RMS, rearrangements leading to the formation of PAX3–FOXO1 or PAX7–FOXO1 fusions are characteristic and have classificatory and prognostic significance [1, 8, 14]. Shern et al. [4] demonstrated that FOXO1-positive tumors are more frequently characterized by CDK4 and MYCN amplifications, whereas in FOXO1-negative tumors alterations of the RAS pathway predominate, including, among others, NRAS, KRAS, HRAS, FGFR4, NF1, and PIK3CA. In the context of head and neck RMS, the MYOD1 mutation is also relevant, being associated with older age, head and neck or parameningeal location, and an unfavorable prognosis [4].

### Locations in the Head and Neck Area. Clinical Division

The head and neck (H&N) region is the site of development for HNRMS in 35% of cases [19].

Clinically, head and neck RMS (HNRMS) is divided into three types. The most common is the parameningeal type, which accounts for 44% of cases. It includes the nasal cavity, nasopharynx, paranasal sinuses, middle ear, infratemporal fossa, and pterygopalatine fossa. The second most frequent type is the orbital type, making up about 25.6% of cases in this location. The third type is the superficial / non-parameningeal type [20]. The prognosis and treatment approach in HNRMS are highly dependent on the tumor’s anatomical location. While the feasibility of complete surgical resection contributes to a favorable outcome, it is clearly not the sole dispositive factor for prognosis. For instance, orbital tumors generally do not require surgical intervention beyond a biopsy, as their treatment is highly effective with non-surgical methods such as chemotherapy and radiotherapy. In contrast, parameningeal RMS is associated with a poor prognosis due to its difficult accessibility, which usually prevents complete surgical removal, and its tendency to invade the skull base. However, the third type, nonparameningeal HNRMS, responds well to radical surgical resection, which not only ensures a better prognosis but can also potentially eliminate the need for subsequent radiotherapy [21] A cohort study involving 20 pediatric and young adult patients demonstrated the critical impact of parameningeal location on the disease course. In this group, 90% (18/20) were parameningeal tumors, mostly diagnosed at advanced TNM stages 3 and 4. Local recurrence in this area carries a very poor prognosis, almost every relapse led to the patient’s death within 11 to 42 months [20].

Poor prognostic factors for HNRMS include parameningeal localization non-radical primary surgical resection, a tumor size exceeding 5 cm, diagnosis at an age greater than 10 years, and a short time to tumor relapse [22].

### Clinical Manifestation Depending on Tumor Location

The clinical presentation of HNRMS is variable and it is linked directly with the tumor’s anatomical origin, its growth rate, and the invasion of adjacent structures [20, 21].

#### Parameningeal HNRMS:

Malignancies developing in the parameningeal spaces grow rapidly and often remain asymptomatic in their early stages, reaching large sizes, which frequently leads to delayed diagnoses [20]. Complicating matters further, the initial clinical signs are often mistakenly attributed to chronic inflammation of the upper respiratory tract. When localized in the nasal cavity, nasopharynx, or paranasal sinuses, the earliest warning signs typically include bloody or purulent nasal discharge. On the other hand, if the primary site involves the middle ear or mastoid region, patients often suffer from a blocked auditory canal and chronic ear discharge [23]. Furthermore, they have a tendency to infiltrate the skull base and spread intracranially [20]. This advanced local spread can present with cranial nerve disorders and other severe neurologic symptoms [23].

#### Orbital HNRMS:

In contrast, orbital HNRMS is usually diagnosed early, which facilitates both rapid diagnosis and fast implementation of therapy [20]. The classic sign of a tumor in this area is a mass on one side that pushes the eye forward, causing proptosis [24]. A clinical examination might further reveal a nodular thickening of one eyelid, exophthalmos that is frequently accompanied by the immobilization of the eyeball, dark bruising around the socket (periorbital ecchymosis), and the onset of strabismus [23].

#### Superficial / Non-parameningeal HNRMS:

Tumors classified as superficial or non-parameningeal, such as those on the cheek, oral cavity, or in the soft tissues of the neck, generally present as fast-growing, visible lumps [23]. As the tumor grows and presses against nearby structures it can cause noticeable facial asymmetry. Furthermore, these lesions can trigger indirect symptoms. For example, if the tumor blocks the Eustachian tube, it can result in the development of serous otitis media [20].

### Diagnostics

The diagnosis of head and neck rhabdomyosarcoma in children is based on a combination of clinical, imaging, and morphological assessment. Nevertheless, in the literature the greatest emphasis is placed on pathomorphological, immunohistochemical, and molecular diagnostics, as well as staging assessment. Owing to the fact that the tumors analyzed are rare, and their clinical and anatomical presentation may overlap with malformations, benign lesions, pseudotumors, and other soft-tissue neoplasms, the possibility of delayed or incorrect diagnosis becomes an important problem in this regard [5, 6]. Thus, an initial diagnosis of an infectious process may occur, followed by the initiation of antibiotic therapy and a delay in extended diagnostic work-up until further tumor growth is observed [8].

After clinical suspicion has been raised, cross-sectional imaging is of fundamental importance, with magnetic resonance imaging being performed, because MRI with multiplanar reconstruction and T1- and T2-weighted sequences before and after contrast administration enables precise delineation of the tumor extent. It should be emphasized that HNRMS is usually isointense relative to muscle on T1-weighted images and hyperintense on T2-weighted images [8]. Moreover, fluorodeoxyglucose positron emission tomography is a technique that allows assessment of glucose uptake by the tumor [25]. Computed tomography, in turn, has limitations in the assessment of RMS margins, because the tumor may enhance similarly to normal muscle; however, it remains useful for evaluating bone destruction and for chest imaging to assess for metastases [8].

Nevertheless, the final diagnosis requires histopathological examination. In this respect, however, morphological assessment alone is not always sufficient. Rodríguez-Vargas and Villanueva-Sánchez [1] emphasized that head and neck RMS in children exhibits cellular heterogeneity, and that the embryonal and alveolar types in particular may resemble other small round cell neoplasms, which necessitates differential diagnosis using immunohistochemistry. Evaluation of a hematoxylin and eosin-stained specimen, supplemented by immunohistochemistry using myogenin, as well as desmin and MyoD1, enables confirmation of the diagnosis and determination of the subtype; however, in some cases additional markers or molecular testing are necessary [1, 3, 6, 15]. Immunohistochemical and molecular diagnostics should be tailored to the suspected subtype.

A complete diagnostic work-up should culminate in the formal determination of disease stage, as risk classification in HNRMS depends not only on the histopathological diagnosis but also on tumour site, maximum tumour diameter, involvement of regional lymph nodes, presence of distant metastases, extent of resection, and FOXO1 fusion status [8, 12]. Staging should be performed in parallel with radiological and histopathological assessment [5]. The most commonly used IRSG system combines pretreatment categories based on tumour location, tumour size, nodal status, and the presence of metastases with a postsurgical grouping system that defines the radicality of resection and the presence of residual disease before the initiation of chemotherapy [8]. First, the primary site of the tumour should be clearly established and assigned to one of the following categories: orbital, parameningeal, or non-orbital non-parameningeal. These categories have prognostic significance and are incorporated into risk-stratification systems [5]. It is essential to determine the maximum tumour dimension, its relationship to the organ or tissue of origin, potential extension beyond the primary site, and its anatomical relationship to critical structures of the head and neck, as tumour size, tumour location, and patient age were included as risk-stratification factors in the EpSSG RMS2005 protocol. An integral component of staging should be the assessment of the IRS group, which reflects the extent of resection and the presence of residual disease after surgical treatment, as well as the evaluation of regional cervical lymph node involvement, which influences allocation to risk groups and prognosis [5, 10]. The staging result should subsequently be interpreted together with the histological and molecular diagnosis, particularly PAX–FOXO1 fusion status and the presence of MYOD1 or TP53 alterations, where assessed. These markers may refine the evaluation of biological risk; however, they do not replace conventional anatomical staging [4, 9].

### Contemporary Therapeutic Strategies in Pediatric Head and Neck Rhabdomyosarcoma

The contemporary management of pediatric rhabdomyosarcoma (RMS) is based on an integrated multimodal model combining systemic chemotherapy with precise local treatment. This strategy is strictly determined by risk stratification, which accounts for histopathological subtype, TNM/IRS staging, and anatomical location. In the head and neck region, orbital locations carry a significantly better prognosis than parameningeal tumors (e.g., sinuses, skull base), which present a substantial therapeutic challenge due to their proximity to intracranial structures [23, 26].

Systemic chemotherapy remains the cornerstone of treatment for all patients, typically utilizing the standard VAC protocol (vincristine, actinomycin D, and cyclophosphamide). Its objective is twofold: the reduction of the primary tumor mass and, crucially, the eradication of micrometastases. In high-risk cases—particularly alveolar rhabdomyosarcoma (ARMS) characterized by *PAX3/7-FOXO1* gene fusions—protocols are often intensified with the addition of irinotecan or etoposide, allowing for a more individualized approach to aggressive tumor phenotypes [23, 27, 28].

The evolution of surgical techniques in otolaryngology has facilitated a shift from mutilating procedures toward organ-preserving surgery. A key role is played by minimally invasive techniques, such as functional endoscopic sinus surgery (FESS) supported by intraoperative navigation, which ensures oncological precision while minimizing trauma to healthy tissues. In scenarios requiring extensive resection, modern reconstructive surgery utilizing free microvascular flaps enables the preservation of airway patency, speech, and swallowing functions, thereby significantly improving the patients’ postoperative quality of life [27, 29].

Radiotherapy serves as a vital adjunct, particularly in parameningeal locations and following non-radical resections. Proton Beam Therapy (PBT) is increasingly becoming the standard of care; by exploiting the Bragg peak phenomenon, it allows for precise dose deposition within the tumor volume with minimal radiation to adjacent tissues. This enables the effective protection of the brainstem, optic nerves, and the hypothalamic-pituitary axis, reducing the risk of late neurotoxic and endocrine complications [30, 31]. The future of RMS treatment lies in the development of immunotherapy and molecularly targeted agents, such as IGF-1R pathway inhibitors, which may offer hope for patients with treatment-resistant disease [28, 32].

## Treatment Complications

Complications of treatment for head and neck rhabdomyosarcoma in children result from the need to use multimodal therapy, including systemic chemotherapy and local treatment in the form of radiotherapy and/or surgery [7, 13, 33]. Of primary importance is its toxicity, as patients are usually treated at a young age, and local interventions involve structures essential for craniofacial growth, the function of the organs of the head and neck, and endocrine development [13, 33].

One of the sequelae of HNRMS treatment is hearing loss. Diepstraten et al. [2] indicate that radiotherapy, proton therapy, brachytherapy within the AMORE framework, defined as Ablative surgery followed by the MOuld technique after-loading brachytherapy and surgical REconstruction. In this framework, brachytherapy is applied as part of the AMORE approach. These modalities, together with surgery, may directly or indirectly affect the nasopharynx, middle ear, nerves, brain, and cochlea, leading to conductive, sensorineural, or mixed hearing loss. In a cohort of 42 survivors of HNRMS who underwent pure-tone audiometry, hearing loss was found in 19.0% according to the Muenster classification, in 14.2% according to CTCAE v4.03, and in 11.9% according to the SIOP classification [2].

Treatment-related complications also affect craniofacial development and the organ of vision. Asakage [16] indicated that potentially permanent and particularly burdensome sequelae of treatment include cataract, retinopathy, growth disturbances, and endocrinological deficiencies. Clinical or radiological dentofacial abnormalities occurred in 80% of patients and included enamel defects, bone hypoplasia and facial asymmetry, trismus, velopharyngeal insufficiency, tooth or root agenesis, and disturbances of root development; bone hypoplasia and impaired root formation were the most common [16]. Graef et al. [11] most frequently observed late local sequelae in the form of bone hypoplasia with facial asymmetry in 40.3% and keratopathy or dry eye in 31.2% of patients. In another study, significantly reduced facial growth was demonstrated in comparison with matched healthy controls, as well as significantly greater facial asymmetry regardless of the local treatment modality used [33].

An important group of complications consists of endocrine disorders, mainly associated with irradiation of the hypothalamic–pituitary region and/or the thyroid gland [13]. In another study in a pediatric population, any endocrine disorder was identified in 35% of patients, with 88% of these cases involving pituitary insufficiency, 6% peripheral thyroid insufficiency, and 6% complex disorders [13]. Growth hormone deficiency was diagnosed in 32% of survivors, and in 39% of this group other pituitary deficiencies coexisted [13].

Zhen et al. [18] in a SEER population-based analysis, demonstrated that the risk of a second malignant neoplasm in patients with RMS was significantly higher than in the general population, with a standardized incidence ratio (SIR) of 1.95, 95% CI 1.44–2.57, *p* < 0.001. The increased risk involved, among others, the bones and joints, soft tissues including the heart, breast, male genital organs, urinary system, brain, and the entire nervous system [18]. In the study by Graef et al. [11], in turn, a secondary neoplasm developed in 4 of 77 patients, that is, in 5.2%.

## Conclusions

Head and neck rhabdomyosarcoma (HNRMS) is the most common pediatric soft tissue sarcoma and constitutes a clinically heterogeneous group of tumors that differ in anatomical location, histopathological subtype, molecular profile, and prognosis.

The anatomical location of the tumor is a key prognostic factor in pediatric RMS. Orbital tumors are associated with the best prognosis, whereas parameningeal tumors have a significantly poorer course due to delayed diagnosis, the limited feasibility of achieving complete surgical resection, and a high tendency to invade the skull base and spread intracranially. Although complete surgical resection, when feasible, contributes to improved local disease control and may improve outcomes in selected patients, it is not the sole determinant of prognosis. Overall prognosis is influenced by multiple factors, including tumor location, histological and molecular characteristics, disease stage, and response to multimodal therapy [24].

Alveolar rhabdomyosarcoma (ARMS) associated with the presence of the PAX3/7–FOXO1 fusion exhibits a particularly unfavorable clinical course, while the embryonal subtype (ERMS) is usually associated with a better prognosis. A correct diagnosis requires a multifaceted approach that includes clinical assessment, modern imaging techniques, histopathological examination, immunohistochemistry, and molecular diagnostics. Magnetic resonance imaging remains the method of choice for assessing local disease progression, while immunohistochemical markers, including myogenin, desmin, and MyoD1, are essential to confirm the diagnosis.

Despite significant therapeutic advances, late complications of treatment remain a serious clinical problem in patients recovered from HNRMS. These include hearing loss, craniofacial growth disturbances, dentofacial abnormalities, ophthalmologic complications, endocrine disorders, and secondary malignancies, which significantly impact patients’ quality of life and require long-term multidisciplinary care.

Current therapeutic strategies focus not only on improving survival but also on minimizing late toxicity and preserving organ function. Individualized multimodal treatment, optimization of local therapy, reduction of radiation exposure to healthy tissues, and the development of molecularly targeted therapies and immunotherapy may further improve treatment outcomes in the future. Due to its anatomical complexity, HNRMS presents a challenge for otolaryngologists, while early recognition and close multidisciplinary collaboration are crucial for timely diagnosis and optimal management.

## Key References

- Krommenhoek, K.B.; Hol, M.L.F.; Slater, O.; Gaze, M.N.; Fennis, W.M.M.; Kolb, F.J.; Smeele, L.; Indelicato, D.J.; Hoogeveen, R.C.; Becking, A.G.; et al. Dental Adverse Effects Following Multimodality Treatment for Head and Neck Rhabdomyosarcoma: Results of a Trans-Atlantic Multicentre Study. Pediatr. Blood Cancer 2025;72. 10.1002/PBC.32004.
  - Presenting crucial data from a large, trans-Atlantic multicenter cohort, this study holds high importance regarding late dental and craniofacial complications in survivors. It provides essential, evidence-based insights necessary for developing guidelines to minimize long-term treatment toxicity and improve the quality of life in pediatric patients.
- Rohde, J.; Henssen, A.; Eggert, A.; Scheer, M. Rhabdomyosarcoma of Head and Neck Varies in Aggressiveness Depending on the Specific Site of Origin. Oral Oncol. 2025;164. 10.1016/j.oraloncology.2025.107263.
  - The observed relationship between tumor site and clinical behavior supports more accurate risk stratification and tailored treatment strategies.
- Graef, S.; DeAngelis, D.; Gupta, A.A.; Wan, M.J. Ocular Manifestations and Long-Term Complications of Rhabdomyosarcoma in Children. Eye (Lond). 2024;38:2907–2911. 10.1038/S41433-024-03175-1.
  - The paper highlights the clinical importance of recognizing and monitoring severe ophthalmic complications associated with multimodal therapy, emphasizing the need for long-term ophthalmological follow-up.
- Patrizi, S.; Vallese, S.; Barresi, S.; Cassandri, M.; Giovannoni, I.; Pedace, L.; Abballe, L.; Vinciarelli, F.; Antonacci, C.; Stracuzzi, A.; et al. Age-Linked DNA Methylation and Gene Expression Patterns in Parameningeal Head and Neck Alveolar Rhabdomyosarcoma Reveal CDK9 as a Promising Therapeutic Target. Pharmacol. Res. 2025;216. 10.1016/j.phrs.2025.107767.
  - This research is highly important because it advances molecular pathology by identifying age-linked epigenetic and gene expression alterations in aggressive parameningeal tumors. By highlighting CDK9 as a novel therapeutic target, it opens new avenues for developing effective molecularly targeted therapies.
- Hebron, K.E.; Odeniyide, P.; Wei, Y.; Gryder, B.E.; Barr, F.G.; Casey, D.L.; Chen, E.Y.; Crompton, B.D.; Dela Cruz, F.S.; Durbin, A.D.; et al. Biological Advances and Current Challenges for Pediatric Rhabdomyosarcoma. Cancers (Basel). 2026;18:888. 10.3390/CANCERS18060888/S1.
  - Synthesizing the most recent breakthrough genomic discoveries and biological insights, this comprehensive review is of major importance to the field of pediatric oncology. It successfully links basic molecular biology directly with modern clinical obstacles, outlining the current translational challenges essential for advancing patient management.
- Vennarini, S.; Colombo, F.; Mirandola, A.; Chiaravalli, S.; Orlandi, E.; Massimino, M.; Casanova, M.; Ferrari, A. Clinical Insight on Proton Therapy for Paediatric Rhabdomyosarcoma. Cancer Manag. Res. 2023;15:1125. 10.2147/CMAR.S362664.
  - This work is crucial because it provides definitive clinical insights into the advantages of Proton Beam Therapy over conventional radiation techniques. It highlights how exploiting the physical properties of protons effectively spares adjacent critical structures, thereby drastically reducing neurotoxic and endocrine complications.

## Data Availability

No datasets were generated or analysed during the current study.
